# Reliability and Criterion Validity of the Assess2Perform Bar Sensei

**DOI:** 10.3390/sports7110230

**Published:** 2019-11-07

**Authors:** George K. Beckham, Danielle K. Layne, Steven B. Kim, Eric A. Martin, Benjamin G. Perez, Kent J. Adams

**Affiliations:** 1 Kinesiology Department, California State University, Monterey Bay, Seaside, CA 93955, USA; dlayne@csumb.edu (D.K.L.); ermartin@csumb.edu (E.A.M.); benperez@csumb.edu (B.G.P.); kadams@csumb.edu (K.J.A.); 2 Mathematics and Statistics Department, California State University, Monterey Bay, Seaside, CA 93955, USA; stkim@csumb.edu

**Keywords:** velocity-based training, accelerometer, autoregulation, inertial measurement unit, resistance training

## Abstract

The Assess2Perform Bar Sensei is a device used to measure barbell velocity for velocity-based training that has not yet been validated. The purpose of this study was to determine criterion validity and reliability of the Assess2Perform Bar Sensei in barbell back squats by comparing it against the GymAware PowerTool, a previously validated instrument. Sixteen injury-free, resistance-trained subjects (eleven males and five females) were recruited. Subjects were tested for their back squat one repetition maximum (1RM). Then, on two separate days, subjects performed two sets of three repetitions at loads of 45%, 60% and 75% 1RM. The GymAware PowerTool and Bar Sensei were attached to the barbell in similar locations for concurrent collection of mean concentric velocity (MCV) and peak concentric velocity (PCV). The Bar Sensei and PowerTool showed generally fair to poor agreement for MCV and PCV when subjects lifted 45% of 1RM (intraclass correlation;ICC 0.4–0.59), and they showed poor agreement when subjects lifted 60% and 75% of 1RM (ICC 0.3–0.4). Inter-repetition/within-set reliability for the Bar Sensei ranged between ICC = 0.273–0.451 for MCV and PCV compared to the far more reliable PowerTool (ICC = 0.651–0.793). Currently, the Bar Sensei is not a reliable or valid tool for measuring barbell velocity in back squats.

## 1. Introduction

In velocity-based training (VBT), practitioners use the velocity of the concentric phase of an exercise as the primary criterion for prescribing a load [1,2], terminating a set [3], or as a marker of fatigue and readiness [4]. Multiple studies have found a strong relationship between various measures of concentric velocity and the percent of one repetition maximum (1RM) of that exercise [4,5,6], as well as a strong relationship between velocity and proximity to failure within a set, demonstrating that the velocity of execution can be used to estimate both the relative and absolute intensity of a given exercise [7,8]. Because the velocity of the bar is a direct reflection of the impulse produced by the lifter against it, and because the load–velocity relationship appears to be reliable, it may serve as a tool that reflects the current level of fatigue in a lifter and their readiness to train at a given time [4]. While coaches often use subjective ratings of effort and velocity in professional practice [9,10], in order to research the topic thoroughly and accurately, direct measures are preferable.

There are a variety of technologies that can be used to measure the velocity of a barbell during training. Linear encoders and linear position transducers, such as the GymAware PowerTool (Kinetic Performance Technologies, Canberra ACT, Canberra, Australia) and Tendo Power Analyzer (Tendo Sports Machines, Trencin, Slovak Republic) are commonly used, but have the drawback that a cable must span between a bar attachment and a stable surface like the ground or squat rack [11]. Inertial measurement units are attached directly to the bar without an anchor and use accelerometers, gyroscopes, and/or magnetometers to measure barbell velocity during resistance training exercises, and transmit this data back to a capturing device such as a computer or tablet, typically via Bluetooth [12]. In comparison to linear position transducers, they have the potential to be used for a greater variety of exercises where the bar may not move in a purely or predominantly linear fashion [12]. Inertial measurement units have grown in popularity due to substantial improvements in accuracy and cost as newer generations of the devices have been developed. Despite these advances and advantages, the accuracy of inertial measurement units is dependent on factors such as accurate calibration of the initial reference frame and mitigation of drift [13]. This makes effective application of the technology in its current evolution as difficult as, if not more than, linear position transducers, particularly with the large variety of possible resistance training exercises.

Several inertial measurement units for resistance training analysis have been evaluated in the literature, such as the Beast wearable device (Beast Technologies, Brescia, Italy) and the PUSH band (PUSH Inc., Toronto, ON, Canada) [12,14,15], though major limitations in the study designs and statistical analyses make their conclusions of good validity and/or reliability questionable (as raised by another recent study [16]). These studies [12,14,15] aggregated kinematic data from different subjects across different reps, sets, and/or loads, resulting in a violation of the statistical assumption of independence of observations needed for correlation and regression analysis [17]. In addition, by aggregating across loads, the range of kinematic values evaluated increased; heterogeneous data samples inflate the correlation between variables, likely resulting in an inflation of the correlation between the device under scrutiny and the device used as the criterion standard [18,19].

The GymAware PowerTool (PowerTool) is a linear position transducer that has been validated [20] and deemed reliable [21] in prior studies for a variety of resistance training exercises. For example, Orange et al. [21] examined the between-session test-retest reliability for back squat repetitions with 20, 40, 60, 80, and 90% of 1RM. They found that peak concentric velocity (highest instantaneous velocity during the concentric phase; PCV) was reliable (ICC ≥ 0.75) for 20, 40, 60% of 1RM (80% and 90% had ICC = 0.68 and 0.65, respectively), and mean concentric velocity (average velocity over the concentric phase; MCV) was reliable for 40, 60, 80, and 90% of 1RM (40–90% of 1RM had ICC > 0.75, 20% of 1RM had ICC = 0.72). Kinetic measures derived from the kinematic outputs of the PowerTool have also shown promise: Crewther et al. [22] found good to strong validity of the PowerTool compared to a force plate in estimating peak force (r = 0.59–0.87) and peak power of squat jumps (r = 0.66–0.90) in loaded conditions. Other recent studies have reported similar findings about kinematic measures obtained from the PowerTool [20,21], establishing it as an adequate choice as the criterion standard for assessing validity of inertial measurement devices.

A relatively new inertial measurement unit, the Assess2Perform Bar Sensei (Bar Sensei), has not yet been evaluated in research to date. The Bar Sensei straps to a barbell using an included jacket, and streams data to an iOS App created by the manufacturer (Assess2Perform, Steamboat Springs, CO, USA). The Bar Sensei reports similar kinematic/kinetic information as other similar devices but has not been tested against previously validated technologies, nor has the reliability of the device been quantified.

Therefore, the purpose of this study was to evaluate the reliability and validity of the Assess2Perform Bar Sensei during the back squat exercise, using the GymAware PowerTool as the criterion device. 

## 2. Materials and Methods

### 2.1. Study Design

The Bar Sensei was compared with the PowerTool to assess criterion validity and reliability in an ecologically valid setting. Males and females with at least 6 months of weekly (>1× per week) back squatting, in a rested state (48 h lower body rest), were recruited to complete a back squat 1RM test at one session, then perform back squats to parallel on two separate occasions at a series of standardized loads meant to represent slow, moderate and fast velocities. The reliability of each device was evaluated within and between each set, and between each testing day. PCVs and MCVs from each device were compared to assess criterion validity. 

Eleven male and five female subjects (age: 22.5 ± 2.6 y, height: 174.1 ± 11.8 cm, weight: 79.5 ± 16.6 kg, back squat 1RM: 114.9 ± 32.3 kg, back squat 1RM divided by body mass: 1.44 ± 0.30, years of squat training: 4.1 ± 3.6 y) were recruited for this study. On the first day of testing, subjects were screened for the following criteria: (i) for the last 6 months, subjects trained squats at least once per week; (ii) subjects had not sustained any injuries in the 6 weeks prior to data collection; and (iii) subjects had not engaged in strenuous lower body exercise in the prior 48 h. This study was approved by the California State University, Monterey Bay Committee for the Protection of Human Subjects (Study Number: CPHS 16-097). All subjects were informed of study procedures and signed informed consent prior to participation.

Subject height and weight were measured using a stadiometer (seca 213 Portable Stadiometer, Seca, Hamburg, Germany) and digital scale (Model ESBS-01, EatSmart Precision Digital, Oakbrook, IL, USA). They reported their age and squat training experience. Subjects reported their current 1RM or the heaviest load they thought they could use for 10 repetitions, which was used to estimate their current back squat 1RM [23]. Prior to warming up, technique standards for the back squat were explained, then subjects performed two sets of five repetitions using a 20 kg barbell to familiarize themselves with the expected squat technique and were coached as necessary to achieve the desired technique by an experienced strength and conditioning coach. Subjects used an approximately shoulder-width stance with toes slightly pointed outwards, squatted to parallel (hip crease at the height of the top of the patella), controlled the eccentric phase and used maximum effort in the concentric phase. Subjects warmed up for 5 min on a cycle ergometer (910Sr Recumbent Magnetic Exercise Bike, Diamondback Fitness, Draper, UT, USA) at 50 watts, pedaling at a cadence of 50–60 RPM. Next, using back squat 1RM testing procedures similar to Vikmoen et al. [24], they squatted 40% of estimated 1RM for 10 repetitions, 75% estimated 1RM for six repetitions, 85% estimated 1RM for three repetitions, and 95% estimated 1RM for one repetition with 2 min rest between each warm up set. Afterward, 2–5% of the estimated 1RM was added to each set and only a single repetition performed, with 3 min rest between each 1RM attempt, until failure occurred. The highest successful load was recorded as the 1RM. After 1RM had been determined, subjects performed 3 sets of 3 repetitions at 25% 1RM for familiarization with the Bar Sensei device. For these familiarization trials, subjects were instructed to control the eccentric phase and told to “perform the concentric phase as fast as possible”. The Bar Sensei device requires a recalibration prior to the initiation of each repetition, necessitating that the subject stand perfectly still for less than 1 s, after which an audible “ding” indicates that the next repetition can start. This process is automated by the device and its software, requiring no input from the user, with a different audible cue indicating that a successful recalibration has occurred (e.g., if the subject stayed still enough for correct calibration), or if the recalibration needs to be completed again.

**Figure 1:**
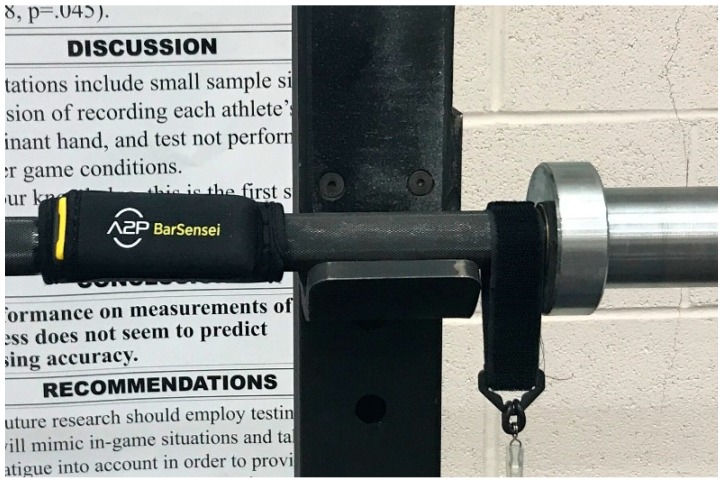
Placement of Bar Sensei and GymAware on a barbell.

Between 3–7 days later, subjects reported back to the laboratory. Subjects were screened to ensure they had not performed strenuous lower body exercise within the prior 48 h. Sessions began with a 5 min warm up on the same cycle ergometer as previously used, again at 50–60 revolutions per minute (RPM) and 50 watts. Subjects then performed parallel back squats for two sets of three repetitions at 45%, 60%, and 75% of 1RM as determined at the initial testing session to represent the broad range of movement velocities that might be experienced in a typical resistance training program. Subjects rested for 2 min between each set. Prior to each set, subjects were reminded to control the eccentric phase and to perform the concentric phase with maximal velocity, and all repetitions were observed by an experienced strength and conditioning coach to monitor squat depth and technique. During this session, both the PowerTool and the Bar Sensei were attached to the barbell (20 kg Ohio Bar, Rogue Fitness, Columbus, OH, USA; Figure 1). For each repetition performed during this session, both MCV and PCV data were collected on each device. Subjects returned again to the lab after this testing session, 3–7 days later, to repeat this protocol.

PCV and MCV values from each device were collected from the iOS App associated with each device (PowerTool: GymAware App v2.2.1; Bar Sensei: A2P Sport App v2.05 and recorded after each set.

### 2.2. Analysis

To evaluate the degree of agreement between the PowerTool and Bar Sensei, a mixed-effects model was used. This model accounts for random effects due to between-subject variability (i.e., subject-specific velocity) and fixed effects due to a potential systematic mean difference between the two measurement devices. Among multiple types of correlation, an intraclass correlation (ICC) is often used for reliability studies [25,26]. Among various kinds of ICC described by Shrout and Fleiss [25], the third kind of ICC (3,k) was chosen because the two devices (Bar Sensei and Powertool) were the only devices of interest in this study with repeated measurements. As demonstrated by Koo and Li [26], the ICC represents the ratio of true variance over the sum of true variance and error variance. To provide robust results without the normality assumption, bootstrapping was used to calculate a confidence interval (CI) for the ICC under the mixed-effects model. One limitation of a correlation is that the magnitude depends on heterogeneity of subjects in the sample [18]. As suggested by Hopkins [18], in addition to the ICC, the typical error (also known as the within-subject standard deviation), the mean difference, and the ratio of standard deviations (SDs) were calculated. In summary, under the mixed-effects model, the three parameters of interest were: ICC between the two devices, the mean difference between the two devices (comparing Bar Sensei to PowerTool) denoted by β, and the ratio of SDs (comparing Bar Sensei to PowerTool) denoted by γ. In addition, ICCs were calculated for the PowerTool and Bar Sensei separately to estimate the ICC among repeated measurements within each device. 

For an ICC, Cicchetti [27] provided guidelines for the degree of agreement between raters (devices): poor if ICC < 0.4, fair if 0.4 ≤ ICC < 0.6, good if 0.6 ≤ ICC < 0.75, and excellent if ICC ≥ 0.75. For the mean difference denoted by β, the null hypothesis was set as H_0_: β = 0, indicating zero mean difference, and the alternative hypothesis was set as H_1_: β ≠ 0. Note that β > 0 is interpreted as an overestimation by the Bar Sensei, and β < 0 is interpreted as an underestimation by Bar Sensei, relative to the PowerTool. For the ratio of SDs denoted by γ = SD_BS_ / SD_PT_, the hypothesis testing was formulated as H0: γ = 1 and H1: γ ≠ 1, where γ > 1 is interpreted as greater variability of measurement error by the Bar Sensei and γ < 1 is interpreted as less variability of measurement error by Bar Sensei, relative to the PowerTool. The significance level for a hypothesis test was fixed at α = 0.05, and the confidence level for a CI was fixed at 1 − α = 0.95.

Bland–Altman plots were used to graphically describe the difference between measurements by the Bar Sensei and the PowerTool [19,28], wherein the y-axis was set as the difference between the two measurements from each device (Bar Sensei measurement minus PowerTool measurement) and the x-axis was set as the average of the two measurements. In the Bland–Altman plots, the degree of disagreement between the two devices was able to be observed with respect to the estimated velocity of an individual subject.

For computations, statistical software R was used with lme4 packages [29,30,31], and the bootMer function in the lme4 package was used to calculate bootstrapped CIs for the parameters of interest [30]. All of the aforementioned analyses were performed separately for each loading condition and velocity measurement (MCV 45%, MCV 60%, MCV 75%, PCV 45%, PCV 60%, and PCV 75% of 1RM).

## 3. Results

The degree of agreement between Bar Sensei and PowerTool seemed fair or poor depending on the level of loading (Table 1). For both MCV and PCV, the degree of agreement tended to decrease as the level of loading increased. According to the guidelines given by Cicchetti [27], Bar Sensei and PowerTool showed fair agreement between MCV and PCV when subjects lifted 45% of 1RM (ICC between 0.4 and 0.59), but they showed poor agreement when subjects lifted 60% and 75% of 1RM (ICC between 0.3 and 0.4). In other words, their agreement was poor when the bar velocity was lower. Table 1 provides an estimated ICC with 95% CI and *p*-value (for testing H_0_: ICC = 0 vs. H_1_: ICC ≠ 0) for each loading and MCV and PCV.

Table 1 also provides an estimated β with 95% CI and *p*-value. For MCV 45%, the speed measured by Bar Sensei was about 0.106 m/s lower than the speed measured by PowerTool on average with 95% CI (−0.131, −0.081). The result was similar for MCV 60%, with an estimated mean difference of −0.094 with 95% CI (−0.118, −0.070). Except for PCV 45%, underestimation of Bar Sensei was statistically significant (i.e., rejection of H_0_: β = 0) for all levels of loading and MCV and PCV. For PCV 45%, an estimated β was 0.009 with 95% CI (−0.022, 0.040).

**Figure 2:**
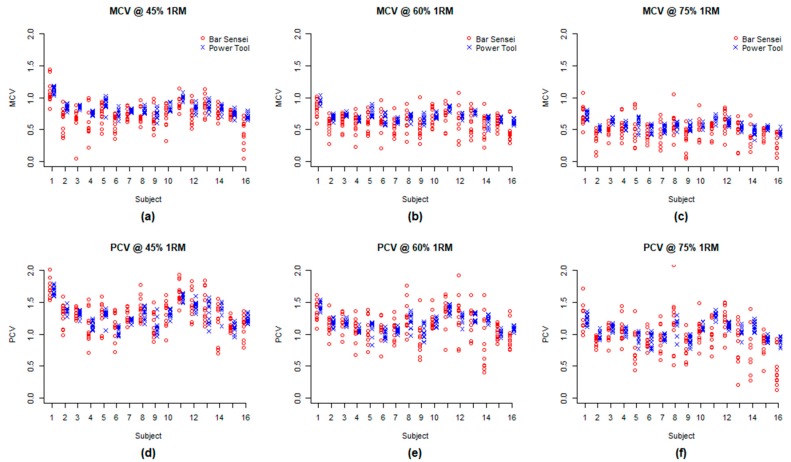
Scatter plot of the measurement by the Bar Sensei and the measurement by the PowerTool side-by-side for each subject for MCV at 45% 1RM (**a**), 60% 1RM (**b**), 75% 1RM (**c**) and PCV at 45% 1RM (**d**), 60% 1RM (**e**), and 75% (**f**).

For all three levels of loading, it was statistically evident that SD_BS_ was greater than SD_PT_ (i.e., rejection of H_0_: γ = 1) for MCV and PCV. For all six comparisons (3 loading conditions and 2 variables), the estimated SD_BS_ was greater by 2.272 to 3.558 times under the mixed effects model. The smallest estimate of γ = SD_BS_ / SD_PT_ was 2.272 with 95% CI (1.974, 2.639) for PCV 45%, and the largest estimate was 3.558 with 95% CI (3.055, 4.099) for MCV 60%. Table 1 provides an estimated γ with 95% CI and *p*-value (for testing H_0_: γ = 1 versus H1: γ ≠ 1) for each loading and MCV and PCV. The greater measurement error by Bar Sensei is also graphically shown in Figure 2. For nearly all subjects and settings, within-subject variation measured by Bar Sensei was greater when compared to the PowerTool. Table 2 contains the calculated ICCs for both Bar Sensei and PowerTool, confirming the observed lower reliability of the Bar Sensei device.

When the typical error (i.e., SD within subject) was estimated without the mixed-effects model, the estimates for MCV were 0.162, 0.162, and 0.160 at three loading levels (45%, 60%, and 75%, respectively) from Bar Sensei, and the respective estimates for PowerTool were 0.056, 0.048, and 0.052, so the respective relative errors were 2.88, 3.40, and 3.06. The respective estimates for PCV were 0.197, 0.220, and 0.248 from Bar Sensei, and the respective estimates for PowerTool were 0.086, 0.070, and 0.070, so the respective relative errors were 2.29, 3.14, and 3.55.

**Figure 3:**
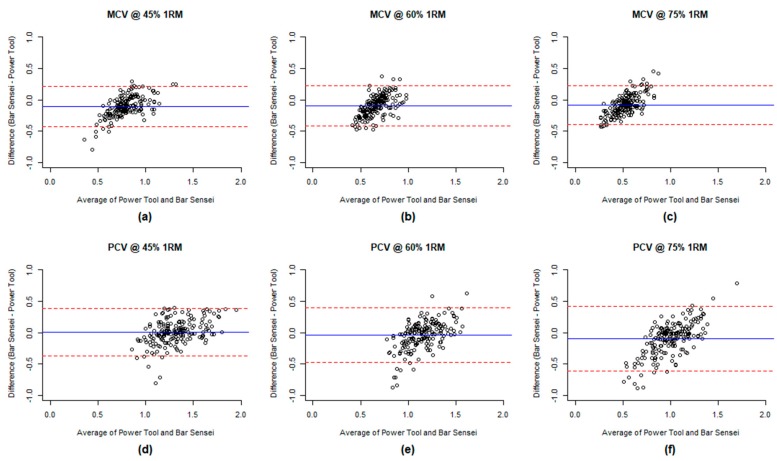
Bland–Altman plot of the measurements by Bar Sensei and PowerTool subject for MCV at 45% 1RM (**a**), 60% 1RM (**b**), 75% 1RM (**c**) and PCV at 45% 1RM (**d**), 60% 1RM (**e**), and 75% (**f**).

The Bland–Altman plot is given in Figure 3 for each loading level and MCV and PCV. On the y-axis, a negative difference implies underestimation by Bar Sensei, whereas a positive difference implies overestimation by Bar Sensei. The plot indicates that underestimation and overestimation by Bar Sensei (relative to PowerTool) depend on the speed of each movement. In particular, it shows that Bar Sensei tended to underestimate the speed of slow movement and overestimate speed of fast movement.

Note that we used the bootstrapping method, which does not require a normality assumption. We further assessed residuals after estimating the model parameters under the mixed-effects model using the original sample, and the distribution of the residuals was symmetric and close to a normal distribution. To this end, if there is any degree of violation of the normality assumption, it would be very mild, and the results from the bootstrapping method seem reasonable.

## 4. Discussion

The primary purposes of this study were to determine criterion validity and reliability of the Assess2Perform Bar Sensei in comparison to the GymAware PowerTool during barbell back squats. Based on the data collected over a range of velocities, sets, and days, we can conclude the Bar Sensei was currently neither a valid nor reliable tool for measuring the peak and mean concentric velocity of a barbell back squat. 

ICCs for the Bar Sensei were generally low to moderate for MCV (ICC = 0.171–0.419) and PCV (ICC = 0.273–0.451), indicating poor reliability. While there are no other studies to the authors’ knowledge specifically evaluating the Bar Sensei, there are other studies evaluating the PUSH device, which is an inertial measurement unit strapped to the forearm during use. These studies have found generally poor reliability in the back squat for the PUSH device [16,32,33], although a major contributing factor could be the placement of the unit on the forearm rather than the barbell itself, as the forearms may move on a path that does not necessarily mimic the path of the barbell.

There are other studies which have evaluated similar technologies to the Bar Sensei, but a frequently made statistical mistake limits their use. For example, Balsalobre-Fernandez et al. [15] compared the Beast device to the SmartCoach Power Encoder (SmartCoach Europe AB, Stockholm, Sweden) (a linear position transducer) during the back squat across a range of loads. To compare between devices, the authors aggregated data from all trials of all subjects before calculating Pearson’s correlation coefficient and calculating the standard error of the estimate. An important assumption of a standard Pearson’s correlation and simple linear regression is the assumption of independence of observations [17], which is violated when repeated observations are added to, but not accounted for, in a dataset. The conclusion that the BEAST sensor is valid and reliable is not supported by their data analyses, given the violation of this important statistical assumption. Furthermore, it is known that correlation and regression analyses are sensitive to the range of data collection, and the correlation can increase when a researcher takes a sample from a heterogeneous population [18,19]. Thus, the statistical approach used in these past studies is likely to elicit inflated r and ICC values, and the correlations cannot fully address the research objective. This erroneous statistical approach has been made in several other validation/reliability studies of similar technologies [12,14,15,34], and this issue has been raised in another recent study [16].

In contrast to the Bar Sensei, we found that the PowerTool demonstrated moderate to good reliability (ICC = 0.651–0.793) amongst the present study population, and could serve as a suitable choice as the criterion measure for velocity during the back squat exercise. These findings are consistent with prior studies of the PowerTool [20,21,33]. Compared to the PowerTool, the Bar Sensei had a generally low agreement and consistency, regardless of the load used. In addition, there was substantially more variability for the Bar Sensei across loads, shown by the roughly three times greater SD (i.e., wider dispersion) for all subjects in Figure 2. The Bland–Altman plots also indicate the Bar Sensei’s tendency to underestimate lower velocity values and overestimate greater velocity values. In total, this indicates that using the Bar Sensei for guidance in terminating a set or modifying the load is inadvisable, given the high variability in measured values.

Across the literature, there are many different ways in which velocity of exercise execution might be used. As some authors have proposed, the individual velocity characteristics of individual repetitions can be used as guidance for when to end a set [3] or whether to increase or decrease the load in subsequent sets [35]. Velocity characteristics have also been proposed as a marker of readiness to train or fatigue [4], for which the peak or average of the velocity variables might be used. The specific application of the velocity information is very relevant to how devices need be evaluated, and it is inadvisable to conclude that a device is generally reliable or valid without certain considerations. For the evaluation of the MCV or PCV of individual repetitions, a design such as that used in the present study is warranted; this study evaluated the reliability of individual repetitions separately across loads. The findings from this study can therefore be generalized to training situations in which individual repetitions are of interest, and can also be applied to situations where the peak or average concentric velocity during a set is of interest, given that the value of the set peak or the set mean is dependent upon the reliability of the individual repetitions. Other studies have evaluated reliability between sets or between days by comparing the mean or peak values from a set or day [16,21]. While this adequately addresses applications where a practitioner is relying on the peak or mean value of a set, it does not necessarily imply that the individual repetitions themselves are also reliable (i.e., the device may have between-set reliability, but not necessarily between-repetition reliability). Thus, there is a need to be clear in future reliability studies as to the extent to which the findings of a study can be applied.

One of the limitations of the present study is that app and algorithm development have both advanced beyond the versions used for the data collection in the present study (A2P Sport is currently v2.22 and GymAware is v2.6). It is possible that newer versions of software have improved the algorithms used for velocity estimation. Thus, future studies should evaluate new versions of software for each device, ensuring that software version information is included in study methods. Additionally, both tools use proprietary software and algorithms that cannot be independently verified for accuracy; future studies could add to the validation of these devices by utilizing an inertial measurement system that provides raw data for more transparent computation and comparison.

## 5. Conclusions

Data from the present study suggest that the version of the Bar Sensei and iOS App used for this study is neither a reliable nor valid tool to measure velocity data while performing barbell back squats. Overall reliability of the Bar Sensei across the loads tested was generally poor. Data across all trials show that the validity of the Bar Sensei, in comparison to the PowerTool, is fair to poor. Reliability and validity were contingent on 1RM intensity. In comparison with the PowerTool, the Bar Sensei appears to underestimate MCV on average in our study population (Table 1). When we closely look at the data via graphic, the Bar Sensei tends to underestimate both MCV and PCV when measuring low velocities and overestimate when measuring high velocities (Figure 3). Continued development of the technology and the iOS Apps for the Assess2Perform Bar Sensei may improve each of these technologies in the future.

## Figures and Tables

**Table 1:** Mixed-effect model to assess the degree of agreement between Bar Sensei (BS) and GymAware (GA): estimated intraclass correlation (ICC), mean difference between the two devices (β), and the ratio of standard deviations (γ) under the mixed-effect model with 95% CI and *p*-value.

Kinematic Variable	Load	Between Device ICC		Mean Difference (β)		Ratio of SDs (γ)	
		Estimate (95% CI)	*p-*Value	Estimate (95% CI)	*p*-Value	Estimate (95% CI)	*p*-Value
MCV	45% 1RM	0.482 (0.267, 0.638)	<0.001	−0.106 (−0.131, −0.081)	<0.001	2.888 (2.480, 3.360)	<0.001
	60% 1RM	0.303 (0.126, 0.471)	<0.001	−0.094 (−0.118, −0.070)	<0.001	3.558 (3.055, 4.099)	<0.001
	75% 1RM	0.329 (0.154, 0.495)	<0.001	−0.081 (−0.105, −0.058)	<0.001	3.319 (2.832, 3.846)	<0.001
PCV	45% 1RM	0.555 (0.335, 0.703)	<0.001	0.009 (−0.022, 0.040)	0.564	2.272 (1.974, 2.639)	<0.001
	60% 1RM	0.362 (0.185, 0.519)	<0.001	−0.037 (−0.071, −0.004)	0.028	3.123 (2.697, 3.581)	<0.001
	75% 1RM	0.361 (0.187, 0.527)	<0.001	−0.099 (−0.136, −0.061)	<0.001	3.425 (2.936, 3.983)	<0.001

The *p*-value for the ICC tests the null hypothesis H0: ICC = 0 versus the alternative H1: ICC ≠ 0.

**Table 2:** Individual reliability of the PowerTool and Bar Sensei.

Kinematic Variable	Load	PowerTool ICC		Bar Sensei ICC	
		Estimate (95% CI)	*p*-Value	Estimate (95% CI)	*p*-Value
MCV	45% 1RM	0.774 (0.594, 0.868)	<0.001	0.419 (0.201, 0.593)	<0.001
	60% 1RM	0.752 (0.548, 0.855)	<0.001	0.171 (0.032, 0.322)	0.010
	75% 1RM	0.651 (0.418, 0.784)	<0.001	0.295 (0.116, 0.471)	<0.001
PCV	45% 1RM	0.793 (0.605, 0.880)	<0.001	0.451 (0.221, 0.619)	<0.001
	60% 1RM	0.775 (0.578, 0.870)	<0.001	0.273 (0.098, 0.440)	<0.001
	75% 1RM	0.761 (0.554, 0.859)	<0.001	0.349 (0.151, 0.529)	<0.001
